# Optimal energy management applying load elasticity integrating renewable resources

**DOI:** 10.1038/s41598-023-41929-1

**Published:** 2023-09-11

**Authors:** Mohamed Mustafa Ragab, Rania A. Ibrahim, Hussein Desouki, Rania Swief

**Affiliations:** 1 Arab Academy for Science, Technology & Maritime Transport, Alexandria, Egypt; 2 Ain Shams University, Cairo, Egypt

**Keywords:** Engineering, Electrical and electronic engineering, Energy harvesting, Energy infrastructure, Renewable energy

## Abstract

Urban growth aimed at developing smart cities confronts several obstacles, such as difficulties and costs in constructing stations and meeting consumer demands. These are possible to overcome by integrating Renewable Energy Resources (RESs) with the help of demand side management (DSM) for managing generation and loading profiles to minimize electricity bills while accounting for reduction in carbon emissions and the peak to average ratio (PAR) of the load. This study aims to achieve a multi-objective goal of optimizing energy management in smart cities which is accomplished by optimally allocating RESs combined with DSM for creating a flexible load profile under RESs and load uncertainty. A comprehensive study is applied to IEEE 69-bus with different scenarios using Sea-Horse Optimization (SHO) for optimal citing and sizing of the RESs while serving the objectives of minimizing total power losses and reducing PAR. SHO performance is evaluated and compared to other techniques such as Genetic Algorithm (GA), Grey Wolf Optimization (GWO), Whale Optimization (WO), and Zebra Optimization (ZO) algorithms. The results show that combining elastic load shifting with optimal sizing and allocation using SHO achieves a global optimum solution for the highest power loss reduction while using a significantly smaller sized RESs than the counterpart.

## Introduction

Smart cities^1,2^ are intended to mitigate energy supply challenges caused by rapid urbanization and population growth, by maximizing efficiency and resource utilization. Electrical energy consumption increased dramatically in recent years, prompting distribution systems to deliver necessary power through proper design and utilization of networks. Incorporating RES^3,4^ such as solar and wind are widely considered to improve grid efficiency and meet demand needs due to their environmental benefits, lower maintenance costs and less environmental impact^5,6^. For these reasons, installing RESs has a significant impact on the distribution system performance since their optimal placement decreases power system’s losses and enhances the voltage profile^7,8^. However, there are uncertainties associated, such as load variations and the random nature of RES^9,10^ which substantially influences the optimization problem’s data and solutions. DSM in smart cities^11,12^ allows customers to regulate their energy usage patterns when incentivized by utilities; in attempt to reduce peak hourly power consumption and minimize the peak-to-average ratio (PAR). There are various strategies to managing the energy consumption and reducing PAR, including shifting of loads^13^.

Numerous optimization algorithms are adopted to handle renewable energies unit’s optimization problems for maximum RES benefits which can be divided into analytical, numerical or Metaheuristic Algorithms (MA). The analytical and numerical techniques are computationally demanding because all possible combinations of RES sites must be evaluated to derive the optimal solution. Furthermore, because the problem is non-linear, the linear programming methods frequently fail to find the optimal solution^14^.

To overcome optimization challenges, MA have gained researchers attention in the past few years^15^, since they can escape local extremum, are unconcerned about initial positions, use parallel iterative searching to solve high-dimensional and multi-objective problems. MAs are commonly divided into two categories: (a) Evolutionary Algorithms and (b) Swarm Intelligence Algorithms. The former utilizes methods inspired by biological evolution such as genetic algorithm (GA) and Jaya Algorithm (JA), while the latter, such as Particle Swarm Optimization (PSO), Firefly Algorithm (FFA), Grey Wolf Optimization (GWO), Zebra Optimization (ZO) algorithm and Sea-Horse Optimization (SHO) algorithms are used to replicate the biological behavior of species^16–23^. Both ZO and SHO are novel MA developed in 2022 demonstrating notable accuracy in achieving high convergence rates while effectively avoiding local extrema. These qualities make them highly promising for addressing complex optimization problems^24^.

Several metaheuristic-based optimization techniques are found in literature^25–35^ and the performance of each technique varies in terms of accuracy and convergence time. Authors in Ref.^25^ proposed bio-inspired algorithms to find the optimal mix of RES units with multi-objectives such as minimizing active power loss, minimizing voltage deviation and maximizing voltage stability index. The study in Ref.^26^ recommended using the Path Finder Algorithm (PFA) to optimally allocate and integrate a PV system. An Improved Crow Search Algorithm (ICSA) based methodology for optimal integration of PV/wind based DGs considering power generation uncertainty and network load demand was proposed in Ref.^27^. In Ref.^29^, authors recommended the application of the honey badger algorithm to evaluate the optimal DG site and size of four RES units. The study in Ref.^30^ employed RESs uncertainty and a β-chaotic sequence spotted hyena optimizer was proposed to allocate two wind turbines for losses reduction, improving voltage profile and stability index. Hybrid optimization techniques have been reported in Refs.^28,31–33^ which exploits the benefits of several optimizers with at a quicker convergence to the optimal solution and an improved the optimization efficiency. An Adaptive PSO (APSO) algorithm-based approach was applied in Ref.^34^ for DG and capacitor bank allocation considering minimization of active and reactive power losses and maximizing the voltage profile. In Ref.^35^, authors reported the optimum RES location and size of a micro electric system with non-stationary power plants using Whale Optimization Algorithm (WOA).

Based on the aforementioned study, several research studies exist to addresses the optimal sizing and placement of DG units, however, incorporating weather-driven sources is difficult due to their uncertainty and intermittency. According to the literature review, the majority of prior work has not considered the fluctuating nature of wind/PV nor load variations in attempt to minimize active losses and voltage deviation, with the exception of Refs.^25,27,30^. The latter took these conditions into account, but at the expense of installing large power units. Moreover, previous research has not considered the use of elastic load shifting strategies in conjunction with optimal DG sizing. Furthermore, to the best of the author’s knowledge, SHO algorithm has not been employed to solve multi-objective optimization problem for allocating and sizing DGs in radial power distribution networks in previous literature.

In this paper an integrated energy management strategy for smart cities has been developed to manage the generation and loading silhouettes while achieving the multi-objective goals of minimizing total active power losses, maintaining bus voltage limits, and lowering the PAR. The proposed framework combines demand side management and elastic load behaviour alongside with renewable energy uncertainty for optimally sizing and siting RESs using a novel SHO swarm intelligence-based metaheuristic approach. The analysis is carried out for an IEEE 69-bus distribution systems using backward/forward sweep for power flow calculation and variation of system loading according to reliability test system (RTS) profile. The main contributions of this paper are summarized as follows:

- A novel metaheuristic-based SHO algorithm is proposed to simultaneously solve a multi-objective and multi-constrained problem for optimizing energy management for citing and sizing RESs.
- A demand side management program is designed to reshape and alter the load profiles under RESs uncertainty which regulates consumption based on precise thresholds derived from statistical features of load profile.
- An elastic load shifting is applied to adjust the PAR to unity as well as reduce active power loss to prevent peaks at valley time while maintaining bus voltage values.
- Compared to other techniques, SHO outperforms GA, GW, WO and ZO algorithms in achieving the highest active power loss reduction yet with smaller sized RESs compared to their counterpart.

The paper is organized into eight sections. An introduction, literature survey, and manuscript objective are represented in “Introduction” section. “Probabilistic model analysis” section, an explanation of the probabilistic models for wind, PV and the load variations is demonstrated. Problem formulation, objective function and technical constrains are explained in “Problem formulation” section. “Applied optimization techniques” and “System under study” sections portrays the optimization algorithm techniques and the proposed system under investigation respectively. “Simulation and results” section depicts the simulation analysis and outcomes. “Discussion” section includes a discussion, along with a conclusion in “Conclusion” section.

## Probabilistic model analysis

Renewable energy sources such as wind and solar power have become increasingly appealing and cost-effective, making them particularly attractive as alternative energy sources in distribution networks due to their primary advantages of low environmental emissions of greenhouse gases^36^. To maximize their utilization in the present study, the distribution system is outfitted with renewable energy sources such as wind and solar. However, the presumption of having constant loads is no more valid due to the great effect of uncertainty and intermittency that appears when using the renewable energy resources. Wind speed and solar irradiation stochastic behavior are commanding the execution of optimal probability load flow where the Probability Distribution Function (PDF) for solar energy and wind energy are taken into consideration.

### Wind and solar data stochastic behaviors

**Figure 1:**
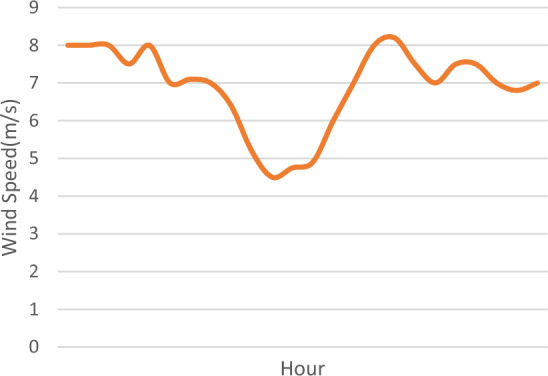
Wind speed hourly forecast^38^.

**Figure 2:**
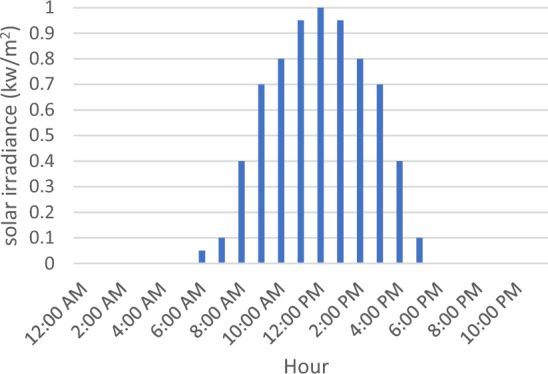
Solar hourly irradiance^38^.

The real hourly wind speed profile and solar irradiance data are shown in Figs. 1 and 2 respectively which can be found in Ref.^37^ or from Willy Online Pty Ltd weather forecast website^38^. Wind turbines and PV modules are assumed to have constant power factor of one.

### Load profile variation

The load variation profile is simulated using a Reliability Test System (RTS) which divides the day into twenty-four intervals (24 h/day)^39^. Table 1 shows the loading percentage relative to average weekday load in percent of daily peak at 24 intervals.

**Table 1:** Hourly peak load in percent of daily peak according to RTS.

Hour	1	2	3	4	5	6	7	8	9	10	11	12
% Of peak load	0.65	0.62	0.59	0.69	0.7	0.61	0.7	0.82	0.92	0.97	0.98	0.98
Hour	13	14	15	16	17	18	19	20	21	22	23	24
% Of peak load	0.96	0.96	0.94	0.93	0.95	0.96	0.96	0.95	0.93	0.89	0.8	0.68

## Problem formulation

Power loss reduction is expected to be the most important goal of power system optimization. In this research, the optimization problem formulation for choosing optimal size and location of wind/PV resources is defined with the purpose of minimising the total active power loss and minimizing PAR in a radial distribution network while satisfying all network and Wind/PV operating and load constraints. By minimizing the system’s peaks, the system’s overall stress will be reduced, hence reducing the need for more generation and distribution capacity.

The search for optimal solution for the multi-objective function (MOF) for this study is calculated using (1):

1 $$MOF= \left({OF}_{1},{OF}_{2}\right),$$

where ${OF}_{1}$ is the first objective function to minimize the total active power losses and ${OF}_{2}$ is the second objective of minimizing the PAR ratio.

The total active power loss can be minimized using (2) and (3):

2 $${P}_{L}=\sum_{i}^{n} {(I)}^{2}\times R ,$$

3 $${OF}_{1}={min}_{\left({P}_{L}\right)}=min \left(\frac{{P}_{L} }{{P}_{LB}}\right),$$

where ${\text{P}}_{\text{L}}$ is the total active power loss in the system, $I$ is the current passing through the system having a resistance $R$, $n$ shows the number of buses and ${P}_{LB}$ is the base power system losses.

PAR represents the shape characteristic of the overall load profile for the whole system and as mentioned earlier, lowering the PAR of the load profile aids the power system energy management by making the grid more stable, efficient, and reliable. This is achieved by increasing spare capacity in the event of a supply shortage if (4) is minimized^40,41^

4 $${OF}_{2}={min}_{\left(PAR\right)}=\left(\frac{{L}_{m}}{{L}_{mean}}\right) = \frac{{L}_{m}}{\frac{{\sum }_{N}Load}{N}},$$

while $N$ represents the total number of time slots in a day, ${L}_{m}$ represent the maximum load value and ${L}_{mean}$ represent the loads mean value.

The optimal wind/PV location and size must satisfy all network and wind/PV operating limitations, including wind/PV size, location, bus voltage limits, and load constraints. In this study, four main technical constraints were considered as follows.

### Wind/PV location constraint

In order to increase system stability, it is assumed that the wind/PV location should be near to the loads. Thus, the Wind/PV location constraint is assumed to start on 2nd bus and can be defined as (5):

5 $${2{\text{nd }}} bus \le {Wind/PV}_{location} \le {n{\text{th}}} bus$$

where ${2{\text{nd }}} bus$ represent bus number two, ${n{\text{th }}} bus$ represent the bus number at IEEE-69 bus system.

### Wind/PV size constraint

Wind/PV sizes and capacity are chosen to be less than or equal to 30% (high penetration level) of the total load power to avoid any possible power outages. This is also chosen to avoid the system’s complete dependability on renewable energy sources due to their intermittent nature. As a result, the capacity of each wind/PV units must fall within the following range (6):

6 $$0\le {P}_\frac{Wind}{PV}\le 0.3 {P}_{T-Loads},$$

where ${P}_{Wind/PV}$ is the total active power from the wind/PV farms and ${P}_{T-Loads}$ is the total load power.

### Voltage bus limit

The amplitude of bus voltage should be limited by the minimum and maximum limits that satisfy the following constrain (7):

7 $${V}_{min}\le {V}_{\left(i\right)}\le {V}_{max},$$

where ${V}_{min}$ and ${V}_{max}$ are maximum and minimum allowable voltages at buses respectively. ${V}_{\left(i\right)}$ is the voltage at the bus $\left(i\right)$*.* ${V}_{min}$ and ${V}_{max}$ are set 0.95 and 1.05 per unit respectively.

### Load elasticity constraint

DSM is crucial in smart grids as it describes strategies used to monitor and regulate the effective use of electric energy at the load and consumer level. Programs for DSM alter the electricity usage pattern in order to achieve desired changes and objectives. The primary objective of DSM is keeping almost a flat energy consumption profile demand thus reducing the energy consumption during peak hours. Consequently, the need to enter new generators with higher production cost is not needed, moreover the production cost will be kept in lower cost levels through operation. DSM also encourages users for less power consumption during peak time and shifting their energy use to off-peak period to flatten the demand load curve.

There are several demand side load management strategies used to alter load shape based on the fact that load can be sensitive to the large cost changes that can be accrued in the electricity market. Load can be classified into shiftable and un-shiftable loads^42^. The proposed methodology in this work controls the un-shiftable load at each hour by setting certain threshold based on the mean and the standard deviation of the load profile to identify the overloaded consumption at peak hours and shifted, “peak clipping”, to the underloaded hours at off peak hours, “valley filling”^43^. Keeping in mind that the objective of the proposed methodology is to reduce the production cost while keeping the PAR near to 1. This flexibility reshapes the daily load profile by smartly controlling the deferrable loads in the context of preserving the main objective function of optimally sizing the RES^44^.

**Figure 3:**
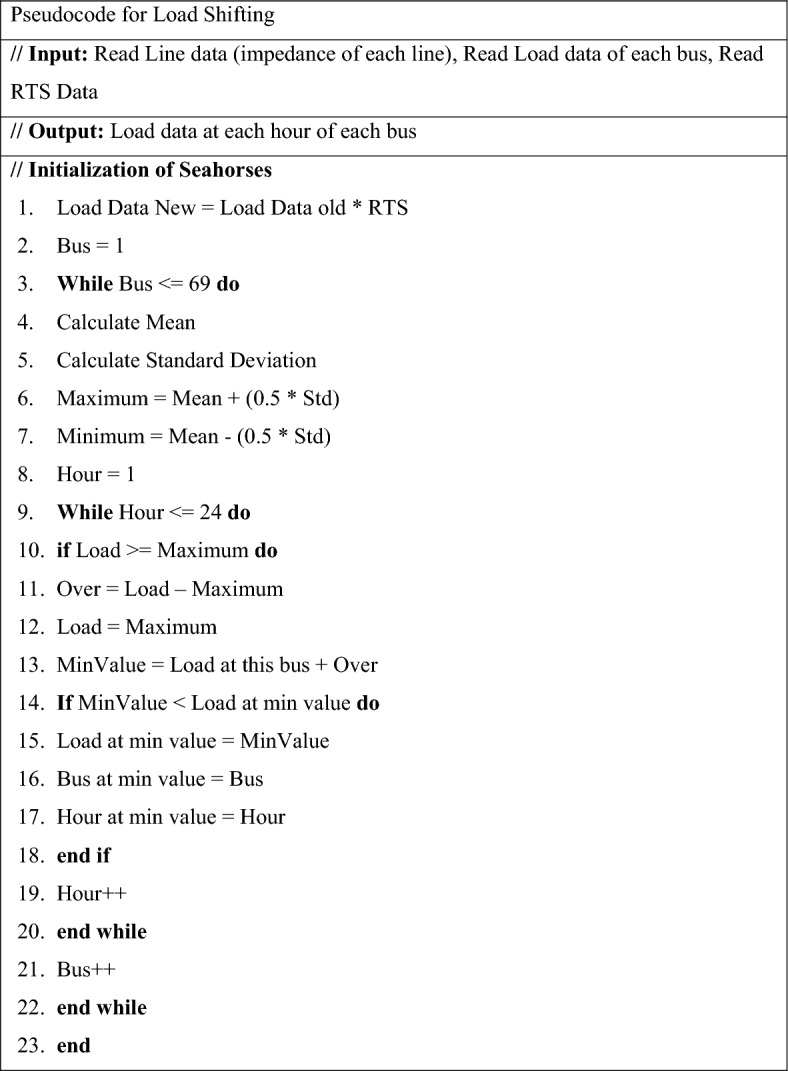
Pseudocode for load shifting.

The proposed load shifting algorithm in this work is selected from the utility viewpoint, to reduce generation costs and reduce the burden on the distribution and utility. Figure 3 shows the pseudocode used in this work which shifts the controllable load such that the load profile is flattened by reducing peaks. It is worth mentioning that this strategy does not change the total energy consumption by the load, in other words, loads are only rescheduled. The range for shifted and non-shifted loads can be represented by (8) and illustrated in the pseudocode of Fig. 3. The idea behind the load shifting strategy applied in this work follows the condition that if the loading percentage exceeds the value of $\left(mean +0.5\times Std\right)$, shifting to off-peak time takes place

8 $${SFL}_{\left(b\right)} \ge mean\left(k\right)+\mathrm{\pounds }\times Std\left(b\right),$$

where ${SFL}_{\left(b\right)}$ is the shifted load at each bus per day, $Std\left(b\right)$ is the standard deviation values at each bus per day (24 h), $mean(k)$ is the average of $(k)$ values at each hour at specific bus, $\mathrm{\pounds }=0.5$ is weight as applied to standard deviation and $\left(b\right)$ is the total values per day at each bus.

## Applied optimization techniques

In this paper, GA and SHO are the two main optimization techniques applied in this work for analysis and testing. GA is considered as a benchmark to validate the efficacy of the sea horse technique in all studied cases. Despite the fact that GA have been in existence for an extended period of time and are widely recognized as a classical optimization technique, their popularity in power system studies persists due to their robustness, adeptness in managing nonlinear complexities, and comprehensive global search capabilities. These attributes are crucial in power systems analysis, particularly in finding optimal solutions in a wide solution space. As previously mentioned, GA is not the only metaheuristic optimization method that have been used in the context of power systems analysis, however, it has been chosen in this work as one of the main comparative techniques due to their popularity and depth of existing literature on their application in power system studies^45^.

### Genetic algorithm

**Figure 4:**
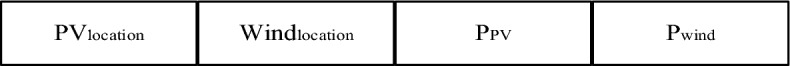
The chromosome for wind/PV units location. ${P}_{PV}$*,* ${PV}_{location}$ represent is the total power output and location of the PV modules respectively, while ${P}_{Wind}$*,* ${Wind}_{location}$ represent the total power output and location of the wind energy system respectively.

A GA is a programming technique that simulates biological evolution based on Darwin’s theory of evolution and survival of the fittest to optimize a population of candidate solutions towards fitness^46^. As seen in Fig. 4, the GA starts to create a random population of chromosomes, that determine the position and size of the scattered generators, which are formed based on the defined constraints of the fitness function. These chromosomes are used to evaluate the fitness function, which presents the distribution system’s total active power losses.

**Figure 5:**
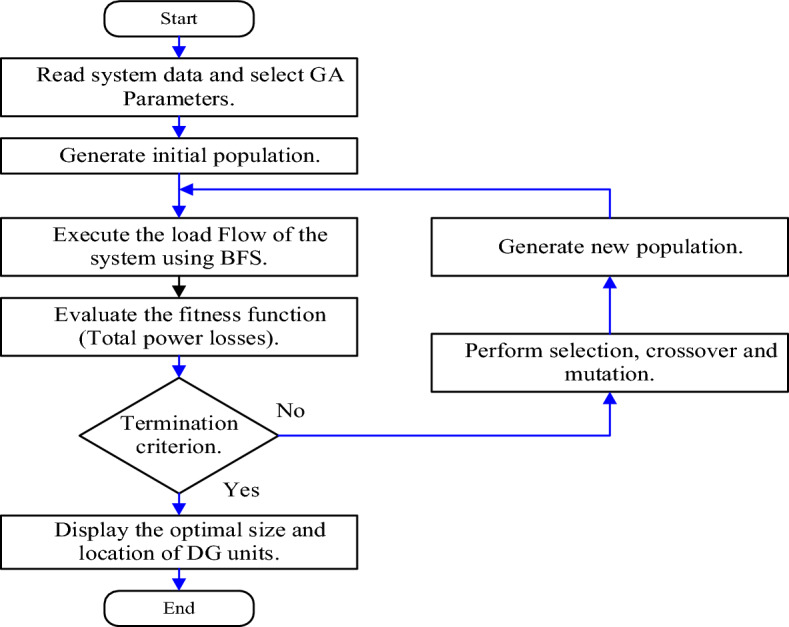
The flow chart of the GA.

The new population is produced based on two operators, crossover and mutation. The main objective of crossover is to search the parameter space. Optimum location for Wind/PV units using the GA flow chart is illustrated in Fig. 5.

Table 2 shows the parameters for the GA algorithm including the mutation probability, crossover probability, the number of the initial population and the maximum number of iterations.

**Table 2:** GA algorithm parameters.

Parameter	Values
Population size	50
Maximum number of iterations	50
Crossover probability	0.8
Mutation probability	0.2

### Sea-Horse Optimization (SHO) technique

Generally, the scientific name for the term “sea horse” refers to a variety of small fish found in warm waters. A brief description of the SHO optimizer is presented in Ref.^24^. SHO is a novel swarm intelligence-based metaheuristic approach consisting of three important components: sea horse movement, predation, and reproduction. To achieve a balance between the exploration and exploitation of SHO, local and global search algorithms for mobility and predation are created, and the breeding behaviour is executed only after the first two behaviours have been performed. The application of SHO to a number of actual engineering problems indicates its great optimization capability and cheap computing cost, paving the way for its future replacement of some existing metaheuristics or other newly suggested algorithms. SHO has a broad range of applications, SHO can be used to solve both discrete and multi-objective optimization problems.

As demonstrated in (9), the SHO implementation process begins by initialising the population by generating a collection of random solutions.

9 $$seahorses= \left[\begin{array}{ccc}{x}_{1}^{1}& \cdots & {x}_{1}^{Dim}\\ \vdots & \ddots & \vdots \\ {x}_{pop}^{1}& \cdots & {x}_{pop}^{Dim}\end{array}\right],$$

where $Dim$ indicates the variable dimension and $pop$ is the population size. SHO exhibits two distinct types of movement: spiral motion and Brownian motion. The spiral motion depends on the upward climb in the three-dimensional components of coordinates $\left(x,y,z\right)$ which can be represented by (10) and (11).

10 $${X}_{new}^{1}\left(t+1\right)={X}_{i}\left(t\right)+Levy\left(\lambda \right)\left(\left({X}_{elite}\left(t\right)-{X}_{i}\left(t\right)\right)\right)\times x\times y\times z+{X}_{elite}\left(t\right),$$

11 $$Levy\left(z\right)=S\times \frac{w\times \sigma }{{\left|k\right|}^{\frac{1}{\lambda }}},$$

where ${X}_{i}\left(t\right)$ represents the new sea horse position after movement at the iteration $t$, ${X}_{elite}$ is the individual, $\left(x,y,z\right)$ are the three-dimensional component axis of coordinates, $Levy\left(z\right)$ is the Lévy flight distribution function for movement, $w$ and $k$ are random numbers selected between [0,1], $\lambda$ is a random number between [0,2], $\sigma$ is a coefficient which depends on random numbers and $S$ is equal to 0.01 as constant number.

The Brownian movement on the other hand depends on the sea waves and drifting actions as demonstrated in (12).

12 $${X}_{new}^{1}\left(t+1\right)={X}_{i}\left(t\right)+rand \times l\times {\beta }_{t}\times \left({X}_{i}\left(t\right)-{\beta }_{t}\times {X}_{elite}\right),$$

where $\mathrm{rand}$ denote the random values [0,1], ${\beta }_{t}$ is the random walk coefficient of Brownian and $l$ is the constant coefficient equal to 0.05.

After movement phase, the predation behaviour starts given that the probability of the sea horse succeeds in capturing food is over 90% as represented by (13).

13 $${X}_{new}^{2}\left(t+1\right)=\left\{\begin{array}{c}{\alpha }^{*}\left({X}_{elite}-rand\times {X}_{new}^{1}\left(t\right)\right)+\left(1-\alpha \right)\times {X}_{elite} if {r}_{2}>0.1\\ \left(1-\alpha \right)\times \left({X}_{new}^{1}\left(t\right)-rand\times {X}_{elite}\right)+{\alpha }^{*}{X}_{new}^{1}\left(t\right) if {r}_{2}\le 0.1 \end{array}.\right.$$

Next is the breeding stage to select the optimum solution where the population is divided into male and female groups based on their fitness levels. Notably, because male sea horses are responsible for reproduction, the SHO algorithm selects half of the individuals with the greatest fitness values as fathers and the other half as mothers. As illustrated in (14)–(16), this division enhances the transmission of positive traits from fathers to mothers for the next generation.

14 $$Fathers={X}_{sort}^{2} \left(1:\frac{pop}{2}\right),$$

15 $$Mothers={X}_{sort}^{2} \left(\frac{pop}{2}+1:pop\right),$$

16 $${X}_{i}^{offspring}={r}_{3}{X}_{i}^{father}+\left(1-{r}_{3}\right){X}_{i}^{mother}.$$

where $Fathers$*, *$Mothers$ are the male and female population respectively, ${X}_{sort}^{2}$ denotes all ${X}_{new}^{2}$ in ascending order of fitness values, ${r}_{3}$ is a random number between [0, 1], ${\text{i}}$ is a positive integer in the range of [1, $\frac{pop}{2}$] and ${X}_{i}^{father},{X}_{i}^{mother}$ represent randomly selected individuals from the male and female populations respectively.

**Figure 6:**
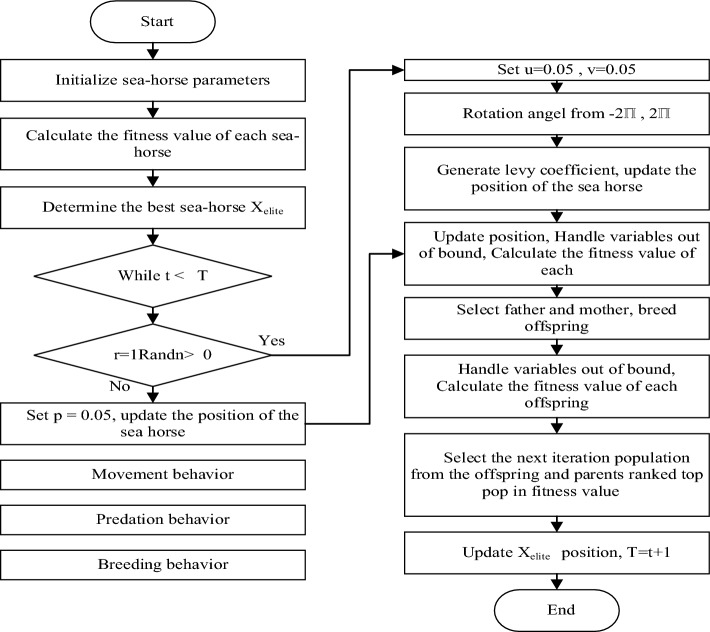
Flow chart of the SHO.

After the sea horse population is updated, offspring breeding takes place. A new population is composed of the offspring and the previous updated sea horses. However, the new population size is $1. 5Pop$. Each individual in the new population is estimated to avoid population expansion without limit. According to fitness values Individuals are sorted from top to bottom in ascending order, and the first $pop$ Sea horses are iteratively chosen as the new population for the next evolutionary process. The flow chart in Fig. 6 depicts this process while the SHO parameters used in this study are found in Table 3, where ${r}_{1}$ and ${r}_{2}$ is the probability of success is kept 0.1.

**Table 3:** SHO algorithm parameters.

Parameter	Values
Population	50
Iterations	50
${r}_{1}$	0.1
Probability of success ${r}_{2}$	0.1

## System under study

This section focuses on a detailed description of using BFS method to calculate the total power losses in the IEEE 69-bus system.

### IEEE-69 bus test system

**Figure 7:**
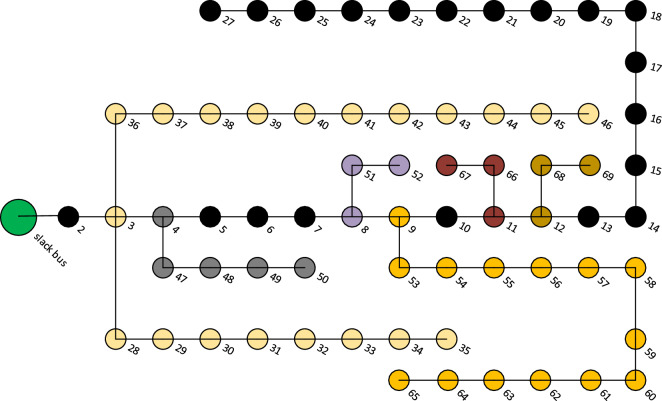
IEEE-69 bus distribution system.

The network employed for testing is the IEEE-69 bus test system which consists of 69 nodes, 5 looping lines, 7 lateral feeders and edges on every branch of the system as represented in Fig. 7. The data of IEEE 69-bus radial distribution test system can be found in Ref.^47^. The total connected loads on this hypothetical system are 3802 kW and 2695 $\mathrm{kVAr}$ respectively with system voltage as 12.66 kV. Bus No.1 (main Sub-station bus) is considered as a slack bus and the remaining buses are considered as load buses.

### Backward/forward sweep method

**Figure 8:**
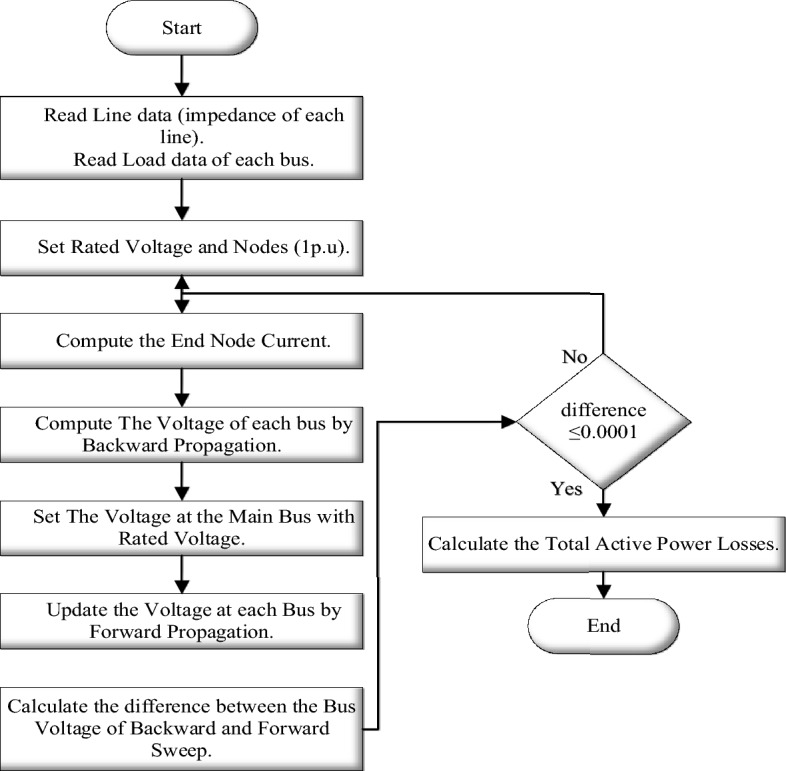
The flow chart of the BFS method.

Numerous techniques, such as conventional and direct load flow (DLF), are available for the analysis of balanced and unbalanced distribution systems. Furthermore, due to convergence issues, traditional load flow techniques such as the Newton–Raphson Method and the Gauss–Seidel Method may become ineffective for load flow studies, rendering them incapable of providing accurate results of line flows and line voltages in the distribution system. The DLF technique, which uses the BIBC (Bus Incidence to Branch Current) and BCBV (Branch Current to Bus Voltage) matrices, is a more robust and efficient method for analysing distribution systems. The IEEE-69 bus test system’s power flow is solved using the BFS Load Flow Algorithm. Reference^48^ provides a brief description of the BFS method. The following flow chart in Fig. 8 describes the operation steps of BFS method.

## Simulation and results

The aim of the study is to keep energy management with high quality for smart cities from electrical point of view. This goal can be achieved by integrating renewable energy resources towards zero carbon city and applying DSM to keep the load profile almost constant, relieving stress on the an electrical utility and reducing power generation, thereby lowering CO2 emissions. The CO2 reduction effect is presented in this paper as reduction in power losses which will lead to emission reduction. The proposed methodology employs an IEEE 69-bus radial distribution system, which is divided into five parts to demonstrate the significance of the study. Part I investigates the impact of RESs with constant power output, by applying the GA and SHO techniques to obtain the optimal allocation for both wind/PV units to minimize power system losses and under normal loading profile. Also, as a side effect of reducing power loss, voltage profile is checked. Part II of the study entails incorporating variable loading profile into the system and determining the optimal wind/PV sources with constant power output. Part III involves taking into account all potential RESs and load uncertainties. In Part IV, all system uncertainties are included with application of elastic loading profile, and two objectives are hibernated to minimize both system losses and PAR.

### Part I: applying GA and SHO techniques with constant wind/PV power and normal load profile

**Figure 9:**
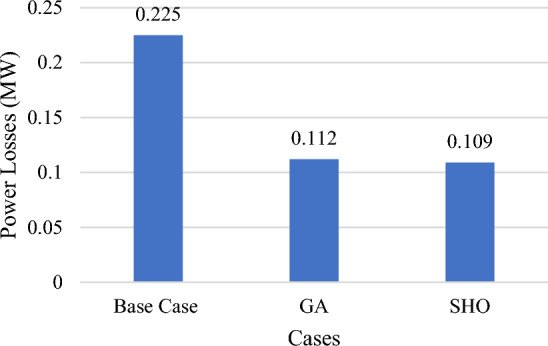
Part I power loss results.

**Figure 10:**
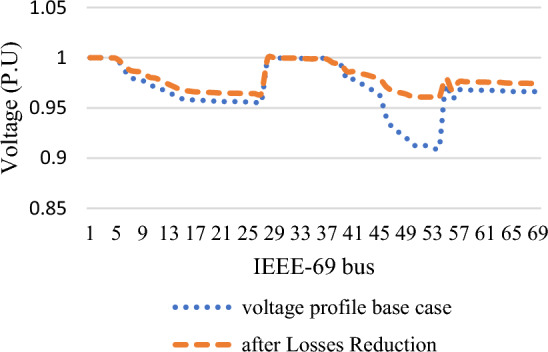
Part I voltage profile curves.

In this part, wind/PV units are installed in the test system with a high penetration level of up to 30% of total system power at a normal loading profile and constant power output from all wind/PV units regardless of irradiance occurrences and wind speed. As indicated in Table 4, the ideal placement of wind/PV in GA case is at bus 22 and bus 50, with total power losses of 112 kW, which is equivalent to a 50.2% decrease in total power losses, using the highest penetration level of 0.80 MW of PV modules and 0.35 MW of wind modules. Using SHO, on the other hand, reveals that the ideal placements for wind/PV units are bus 50 and bus 52, with total power losses of 104.75 kW, representing a 53.5% reduction in total active power losses as shown in Fig. 9. Consequently, 0.75 MW of PV modules and 0.34 MW wind farm sizes are selected, with a penetration level equal to 28.6%. Figure 10 shows the voltage profile improvement obtained before and after reducing the total system power losses.

**Table 4:** Part I results obtained by GA and SHO.

Method	Optimal location (bus number)	PV size (MW)	Wind size (Mw)	Power loss (MW)	Penetration level	Loss reduction
Base case	–	–	–	0.225	–	–
GA	22,50	0.80	0.35	0.112	30%	50.2%
SHO	50,52	0.75	0.34	0.1047	28.6%	53.5%

### Part II: applying GA and SHO techniques with constant wind/PV power and variable loading percentage using RTS

**Figure 11:**
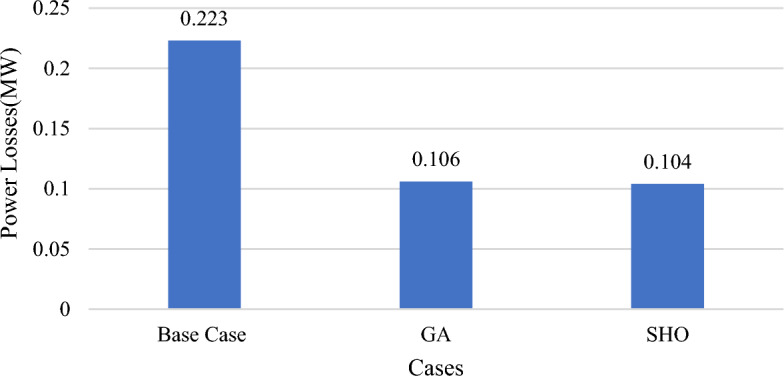
Part II power loss results for GA and SHO.

In this part, wind/PV units are installed to the test system at variable loading profile without applying DSM and constant power output from all wind/PV units regardless of irradiance occurrences and wind speed. The total power losses for the tested base case is equal to 223 kW as indicated in Table 5. Using GA and SHO algorithm, the ideal placement of wind/PV in GA case is at bus 12 and bus 50, with total power losses of 106 kW, which is equivalent to a 52.4% decrease in total power losses with a penetration level of 4.5%. This was accomplished with 0.108 MW of PV modules and 0.061 MW of wind turbines. Using SHO, however, reveals that the ideal placements for wind/PV units are bus 17 and 50, with total power losses of 104 kW as shown in Fig. 11, which represents a 53.3% decrease in total active power losses. As a result, 0.0343 MW of PV modules and 0.1095 MW wind farm size with a penetration level equal to 3.7% are required.

**Table 5:** Part II results obtained by GA and SHO.

Method	Optimal location (bus number)	PV size (MW)	Wind size (Mw)	Power loss (MW)	Penetration level	Loss reduction
Base case	–	–	–	0.223	–	–
GA	12,50	0.108	0.061	0.106	4.5%	52.4%
SHO	17,50	0.0343	0.1095	0.104	3.7%	53.3%

### Part III: applying GA and SHO techniques with all system uncertainty

**Figure 12:**
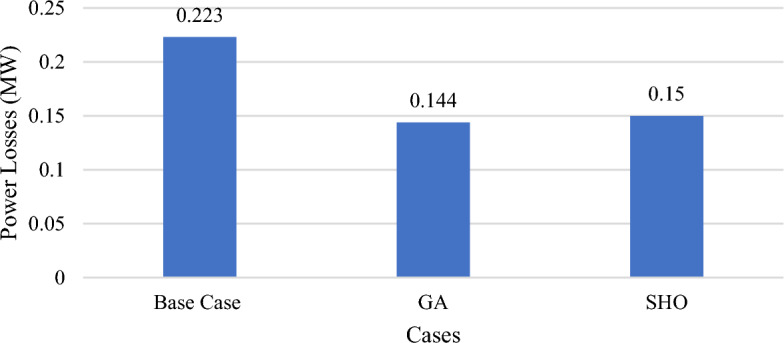
Part III power loss results for GA and SHO.

In this part, wind/PV units and the load variability using RTS are applied to the test system at variable power output from all wind/PV units including irradiance occurrences and wind speed fluctuations without applying DSM. The total power losses for the tested system equal 223 kW as indicated in Table 6 and utilizing the GA and SHO algorithm. GA algorithm results reveal that the ideal placement of wind/PV is at bus 50 and bus 53, with total power losses of 144 kW, corresponding to a 35.4% decrease in total power losses. This has been achieved at a penetration level of 0.80 MW and 0.35 MW for wind/PV modules respectively. On the other hand, applying SHO reveals that the ideal placements for wind/PV units are bus 19 and bus 50, with total power losses of 150 kW as shown in Fig. 12, which represents a 32.7% decrease in total active power losses. Consequently, 0.75 MW of PV modules and 0.34 MW wind farm size with a penetration level equal to 28.6%.

**Table 6:** Part III results obtained by GA and SHO.

Method	Optimal location (bus number)	PV size (MW)	Wind size (Mw)	Power loss (MW)	Penetration level	Loss reduction
Base case	–	–	–	0.223	–	–
GA	50,53	0.80	0.35	0.144	30%	35.4%
SHO	19,50	0.75	0.34	0.150	28.6%	32.7%

### Part IV: applying GA and SHO techniques with system uncertainty and applying load elasticity

**Figure 13:**
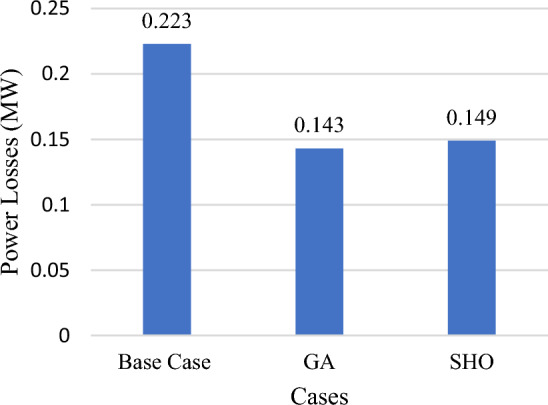
Part IV power loss results.

**Figure 14:**
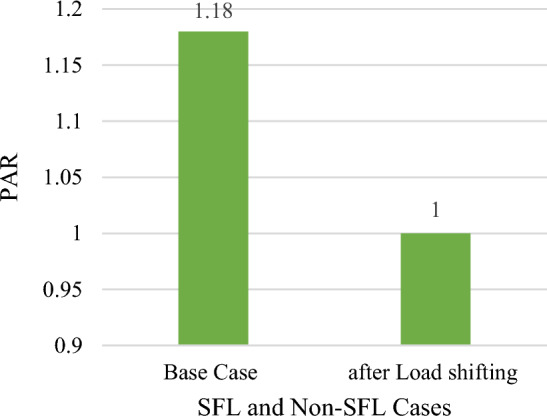
Part IV PAR results.

**Figure 15:**
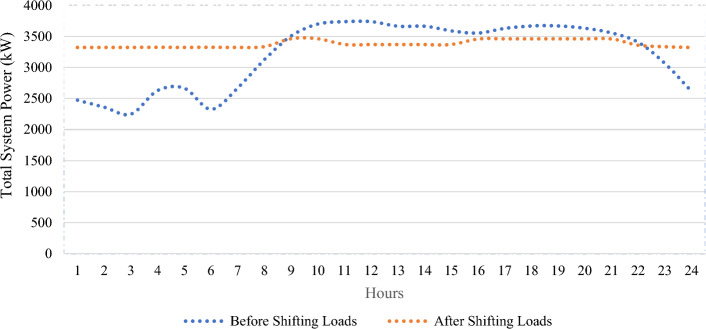
Total power profile applied to IEEE-69 bus system before and after load shifting.

**Figure 16:**
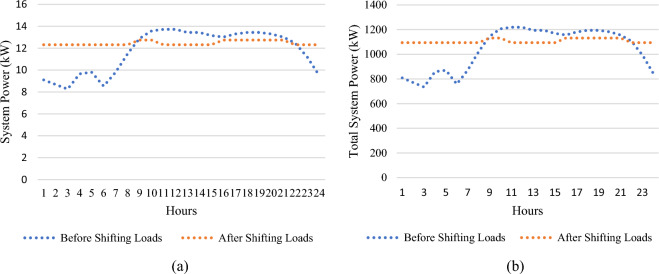
Active power profile before and after load shifting (**a**) Bus Number 26, (**b**) Bus Number 50.

Variable power output from all wind/PV units and the load variability are applied to the test system in this section, with improvements to the system load profile by applying load elasticity. According to Table 7, The total power losses for the tested system are 223 kW, the ideal placement of wind/PV in the GA case is at bus 50 and bus 53, with total power losses of 143 kW, which is equivalent to a 35.8% decrease in total power losses. This has been accomplished at a penetration level of 0.80 MW and 0.35 MW for PV and wind units respectively. Using SHO technique, however, total power losses of 149 kW are achieved, representing a 33.2% reduction in total active power losses, as shown in Fig. 13 with ideal placements for wind/PV units at buses 50 and 53. As a result, 0.44 MW of PV modules and 0.35 MW wind farm size are required, with a penetration level equal to 20.7%. Figure 14 depicts the total PAR reduction before and after the DSM technique was applied. It is worth noting that after implementing the load shifting strategy, the PAR values have been reduced to unity. PAR reduction is important in lowering load demand fluctuations, decreasing electricity bills, and reducing the requirements of constructing new conventional power plants. In addition, Fig. 15 shows the daily power profile for the IEEE-69 bus system before and after applying load shifting methodology. Using the proposed strategy resulted in a smooth power profile for all buses. Furthermore, the power profile for bus 27 has been evaluated and shown in Fig. 16a since it represents the farthest bus and the highest in terms of active power losses. Moreover, bus 50’s profile is depicted in Fig. 16b as it represents the highest load among all system buses. As seen from both Fig. 16a,b, the proposed strategy has been able to maintain constant power values in both extreme cases with lower power fluctuations.

**Table 7:** Part IV results obtained by GA and SHO.

Method	Optimal location (bus number)	PV size (MW)	Wind size (Mw)	Power loss (MW)	Penetration level	Loss reduction	PAR reduction
Base Case	–	–	–	0.223	–	–	–
GA	50,53	0.80	0.35	0.143	30%	35.8%	17%
SHO	50,53	0.44	0.35	0.149	20.7%	33.2%

## Discussion

This paper presented an energy management strategy that combines the beneficial features of optimally sizing and allocating RESs and DSM strategies applied for smart cities. The proposed strategy was applied using the SHO algorithm in case of load and RESs uncertainty. Results were compared in previous sections with GA for four different scenarios to test its validity and investigate the system performance.

### Analysis of results of the four investigated scenarios

Table 8 summarizes results from Part I to Part IV. Part I used GA and SHO to determine optimal RESs sizing and location in the absence of load and resources uncertainty. It is clear from Table 8 that adopting SHO algorithm produces better results than GA in terms of achieving lower total power losses. For results of Part II and after applying load variability at constant PV/wind power, the SHO algorithm still outperforms GA in terms of better loss reduction while using smaller sized RESs. However, after accounting all system uncertainty (load variability, wind/PV uncertainty), Part III results show that GA achieved lower power losses than its counterpart. Finally, in Part IV, after applying all-system uncertainties and DSM load shifting from on-peak periods to off-peak periods, not only is there a significant reduction in power loss, but also reduction in system PAR. Although GA achieves a higher loss reduction percentage by 0.4% than SHO, the difference is offset by installing 9.3% smaller RES and almost half the PV installation capacity in SHO.

**Table 8:** Summary of results from part I to part IV.

Case	P (MW) (PV, wind)	P (MW) (PV, wind)	Power losses (MW)	Power losses (MW)	Penetration level %		PAR old	PAR new
	GA	SHO	GA	SHO	GA	SHO		
Base case	–	–	0.22493/0.22353		–	–	1.2	1.2
Part I	0.80, 0.35	0.75, 0.34	0.112	0.1047	30%	28.6%	1.2	1.2
Part II	0.108, 0.061	0.0343, 0.109	0.106	0.104	4.5%	3.7%	1.2	1.2
Part III	0.80, 0.35	0.75, 0.34	0.144	0.150	30%	28.6%	1.2	1.2
Part IV	0.80, 0.35	0.44, 0.35	0.143	0.149	30%	20.7%	1.2	1

### Comparison with previous studies and other metaheuristic algorithms

**Figure 17:**
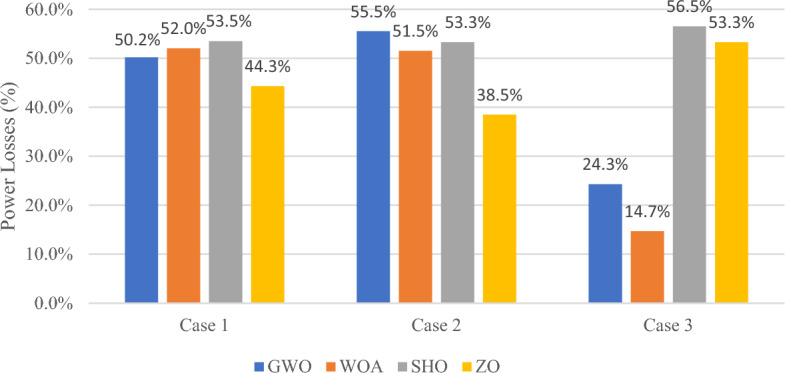
Power loss percentage results of GWO, WO, ZO and SHO algorithms.

In published research, a wide variety of strategies for optimally selecting the location and size of RES are presented^49^. The strategy of living organisms when hunting and trapping prey has been the main idea of various metaheuristic algorithms with the GWO and WO algorithms being two of the most prominent techniques involved in addressing the optimization problem for sizing and placement of DGs. Furthermore, the ZO is a newly developed algorithm that appears to be as a recent rival to bio-inspired algorithms. In this section, the SHO algorithm performance capabilities are tested in comparison with the GWO, WO, and ZO algorithms to prove the accuracy of the SHO algorithm in solving multi-objective optimization problem such that three cases where investigated. Case 1 compares the performance of GWO, WO, ZO and SHO techniques with constant wind/PV and load profile; Case 2 examines the effectiveness of integrating the aforementioned algorithms with constant wind/PV power and variable loading percentage using RTS; and Case 3 investigates the performance of the 4 algorithms when all system uncertainty are taken into account. The backward forward sweep power flow method has been utilized to calculate system variables, such that the main objective is to minimize the power loss and maintain bus voltage limits. Table 9 explores the performance of the SHO and GW, WO and ZO for the three cases and the chart in Fig. 17 summarizes the power loss results.

**Table 9:** Comparison results between WOA, GWO, ZO and SHO.

Case	Method	Optimal location (bus number)	PV size (MW)	Wind size (Mw)	Power loss (MW)	Penetration level	Loss reduction
Case 1	Base case	–	–	–	0.223	–	–
	GWO	21,50	0.8	0.34	0.111	30%	50.2%
	WOA	17,53	0.8	0.30	0.107	29.5%	52%
	SHO	50,52	0.75	0.34	0.104	28.6%	53.5%
	ZO	40,50	0.63	0.34	0.124	28.6%	44.3%
Case 2	GWO	50,53	0.8	0.34	0.100	30%	55.3%
	WOA	50,53	0.8	0.25	0.108	27%	51.5%
	SHO	17,50	0.0343	0.1095	0.104	3.7%	53.3%
	ZO	50,53	0.46	0.170	0.137	16.8%	38.5%
Case 3	GWO	50,53	0.8	0.34	0.169	30%	24.2%
	WOA	2,9	0.8	0.2	0.190	26.3%	14.7%
	SHO	50,53	0.8	0.34	0.098	30%	56.5%
	ZO	51,53	0.8	0.34	0.104	30%	53.3%

In case 1, the analysis focuses on the system’s performance under constant RES generation by conducting a comparison among the four algorithms. The results presented in Table 9 show that SHO achieved minimum active power losses, followed by the WO, GWO, and ZO algorithms, with corresponding loss reduction of 53.5%, 53.5%, 52% and 43.5% respectively.

Case 2 explores the performance of the four algorithms with RES generation uncertainty. In case 2, the GWO managed to achieve the best objective function value, followed by SHO, WOA and ZO as recorded in Table 9 such that the loss reduction was 55.3%, 53.3%, 51.5% and 38.5% respectively.

Considering all system uncertainties for generation and load profile in case 3, the SHO algorithm demonstrated superior performance compared to its counterpart, achieving 56.5% loss reduction while maintaining same generation capacity and penetration level as GWO and ZO algorithms as shown in Table 9.

**Figure 18:**
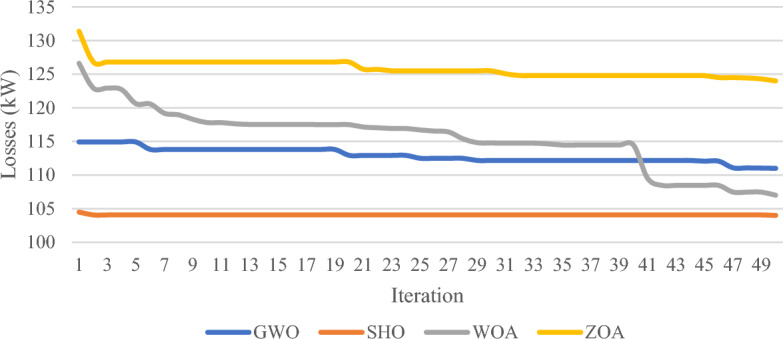
Convergence plots for GWO, WO, ZO and SHO algorithms.

Figure 18 depicts the convergence plot and Table 10 indicates the convergence time and iterations acquired by each algorithm for case 1, showcasing the SHO method’s performance in reaching the optimal solution for case 1, as compared to the WO, GWO and ZO techniques. Notably, the SHO not only attained a global minimum among the four algorithms but also demonstrated fast convergence compared to GWO and WO, requiring the least number of iterations to reach the optimal solution.

**Table 10:** Comparison results of convergence times of WOA, GWO, ZO and SHO.

Algorithm	Convergence time (s)	Iterations
GWO	14.89	50
WO	17.28	47
SHO	11.59	2
ZO	11.36	50

Referring to Fig. 17, it is apparent that SHO surpasses GW, WO and ZO algorithms in both case 1 and 3, achieving a higher percentage reduction in power loss. Although GWO performs better than SHO in case 2 by 2% reduction difference, it is important to note that SHO still outperforms all other algorithms in terms of number of iterations required to reach the optimal solution as demonstrated in Table 10.

### Demand side management evaluation

This work focuses on implementing a demand side management program by limiting load energy consumption during peak hours and measuring PAR as a metric to determine the efficacy of the DSM strategy^50^. Referring to Fig. 15 and the total power profile applied to IEEE-69 bus system before load shifting, it can be observed that a peak demand of 3738 kW is exhibited during the morning early hours. In addition, during the time of the day between the hours of 9 to 22, the load demand surpasses the average load demand, and the PAR values were 1.2. In this case, when the consumer demand exceeds that provided by the average power provided by the utility, utility company will tend to utilize more power plants to fill the gap between generation and consumption. Shifting loads from peak to off-peak periods will not only help in reducing the operational costs associated with the addition of new power plants, but it will also help in reduction of carbon emissions and a reduced electricity bill from the consumer viewpoint. By employing the load shifting strategy used in “Problem formulation” section, PAR is maintained unity and consequently the cost of energy will be reduced. Table 11 shows the initial load and the modified load demand after demand response and hourly increase or decrease in load demand in percentage each hour during the day.

**Table 11:** Hourly load demand before and after load shifting.

Hour	Before load shifting (kW)	After load shifting (kW)	%	Hour	Before load shifting (kW)	After load shifting (kW)	%
1	2472.424	2790.246606	1.128546967	13	3663.102	3369.149	0.919753
2	2359.358	2789.686606	1.182392246	14	3664.102	3368.028	0.919196
3	2246.2921	2790.242397	1.242154748	15	3589.059	3368.018	0.938413
4	2627.511	2791.417606	1.06238094	16	3552.0367	3460.163831	0.974135158
5	2666.533	2790.246606	1.046394928	17	3629.0805	3460.163831	0.953454692
6	2325.336	2791.704606	1.200559664	18	3668.102	3460.164	0.943312
7	2668.533	2790.194606	1.045591194	19	3669.102	3460.164	0.943055
8	3125.796	3333.026	1.066296713	20	3632.081	3460.164	0.952667
9	3507.015	3462.71	0.987367	21	3557.037	3460.164	0.972766
10	3698.124	3462.548	0.936298	22	3405.949	3357.294	0.985715
11	3737.146	3369.149	0.90153	23	3064.752	3333.556	1.087708239
12	3738.146	3369.149	0.901289	24	2609.489	2788.756606	1.068698357

## Conclusion

The integration of RESs and their optimal utilization is one of the crucial topics for the construction of smart cities. Furthermore, owing to a significant amount of RES penetration and integration in power networks, power system planning cannot neglect uncertainties in generation and load behavior. This paper proposed a multi-objective optimal power resource management strategy combined with DSM and elastic load profile. The optimal installation location and sizing of RESs are determined taking into account minimization of power losses and reduction of PAR to achieve lower production and generation costs. A novel Meta-heuristic SHO was applied to handle the multi-decision problem and the performance of the proposed methodology was tested on an IEEE-69 test bench system for four different scenarios compared to GA as a benchmark algorithm. First, the results illustrated total power losses reduction using SHO compared to GA in the case of constant generation and load. Second, the simulation results confirmed the superiority of SHO over GA in the case of load uncertainty and constant generation. Third, the results were verified in case of load and RES intermittency followed by a fourth scenario in the presence of load and sources uncertainty, as well as applying flexible load shifting from peak to off-peak times. The load shifting strategy manages the fixed load consumption during each hour by setting thresholds based on mean and standard deviation on load profile to identify instances of excessive consumption during peak hours and redistribute the load to off-peak. Simulation results validate that the performance of the proposed SHO algorithm was advantageous in substantially reducing the total power losses, maintaining bus voltage profile within permissible limits, reducing the PAR to unity thus avoiding any power fluctuations, yet with small RES size. The effectiveness of the SHO algorithm was tested against other recent MA algorithms in literature for three different test cases, which showed that the SHO achieved the best optimal in the majority of cases compared to those achieved by GWO, WO and ZO algorithms respectively. When compared to other algorithms, SHO results demonstrated higher accuracy and an acceptable convergence time with the least number of iterations.

## Data Availability

The data analysed during the current study available from the corresponding author on reasonable request.

## List of symbols

BFS: Backward forward sweep
BIBC: Bus incidence to branch current
BCBV: Branch current to bus voltage
DG: Distributed generation
DSM: Demand side management
DLF: Direct load flow
*Dim*: Dimension of the variable
GA: Genetic Algorithm
GWO: Grey Wolf Optimization
ICSA: Improved Crow Search Algorithm
JA: Jaya Algorithm
$${\text{kW}}$$: Kilowatt
$$SFL$$: Shifted load
$Std$: Standard deviation values
$$S$$: Constant number = 0.01
WOA: Whale Optimization Algorithm
ZO: Zebra Optimization Algorithm
$B_{old}$: Previous load data
$B_{new}$: Updated load data
$$\begin{gathered} Fathers \hfill \\ Mothers \hfill \\ \end{gathered}$$: Male and female population
$$I$$: Current (Ampere)
$$L_{m}$$: Maximum load (W)
$L_{mean}$: Loads mean value (W)
$$l$$: Constant coefficient = 0.05
$N$: Total number of time slots in a day
$n$: Number of buses
$n{\text{th}} bus$: Bus number at IEEE-69 system
$OF_{1}$: Objective function to minimize total active power losses
$OF_{2}$: Objective function to minimize peak to average ratio
$P_{L}$: Total active power losses (W)
$P_{LB}$: Total base active power losses (W)
$$P_{Wind/PV}$$: Total $$\left( {{\text{Wind}}/{\text{PV}}} \right)$$ power (W)
$P_{T - Loads}$: Total load power (W)
$P_{PV}$: Total power output from PV
MA: Metaheuristic Algorithms
MOF: Multiple objective functions
$mean$: The average values
PSO: Particle Swarm Optimization
PFA: Path Finder Algorithm
*pop*: Population size
PAR: Peak to average ratio
PDF: Probability density function
PV: Photovoltaic
RTS: Reliability test system
RES: Renewable energy resources
SHO: Sea-Horse Algorithm
$P_{Wind}$: Total power output from wind
$$PV_{Location}$$: PV location
$$R$$: Bus resistance (Ω)
${\text{r}}_{1} ,{\text{r}}_{2} ,{\text{r}}_{3}$: Random number between [0, 1]
$$u,p,v$$: Constant parameters
$V_{min}$: Min. voltage allowance (per unit)
$V_{max}$: Max. voltage allowance (per unit)
$$Wind_{Location}$$: Wind location
$$Wind/PV_{location}$$: $${\text{Wind}}/{\text{PV}}$$ location
$$w,k$$: Random numbers
$X_{new}^{1} \left( t \right)$: New position of the SHO
$$\begin{gathered} X_{i}^{father} \hfill \\ X_{i}^{mother} \hfill \\ \end{gathered}$$: Random individuals selected from male, female populations
$X_{i} \left( t \right)$: Sea horse new position
$X_{sort}^{2}$: $\left( {\text{X}} \right){\text{2new}}$ In ascending order of fitness values
$\left( {x,y,z} \right)$: Three-dimensional component axis of coordinates
$\pounds$: Weight applied to standard deviation
$\alpha$: Adjusted moving step size
$\lambda$: Random number between [0,2]
$$\sigma$$: Coefficient
$\beta_{t}$: Random coefficient of Brownian
$2{\text{nd}} bus$: Bus number two

## References

[1] Kim JH (2022). Smart city trends: A focus on 5 countries and 15 companies. Cities.

[2] Razmjoo A, Gandomi AH, Pazhoohesh M, Mirjalili S, Rezaei M (2022). The key role of clean energy and technology in smart cities development. Energy Strategy Rev..

[3] Haegel N, Kurtz S (2021). Global progress toward renewable electricity: Tracking the role of solar. IEEE J. Photovolt..

[4] Serban AC, Lytras MD (2020). Artificial intelligence for smart renewable energy sector in Europe—Smart energy infrastructures for next generation smart cities. IEEE Access..

[5] Hannan MA, Al-Shetwi AQ, Ker PJ (2021). Impact of renewable energy utilization and artificial intelligence in achieving sustainable development goals. Energy Rep..

[6] Silva C, Faria P, Vale Z, Corchado JM (2022). Demand response performance and uncertainty: A systematic literature review. Energy Strategy Rev..

[7] Purlu M, Turkay BE (2022). Optimal allocation of renewable distributed generations using heuristic methods to minimize annual energy losses and voltage deviation index. IEEE Access..

[8] Ali A, Keerio MU, Laghari JA (2021). Optimal site and size of distributed generation allocation in radial distribution network using multi-objective optimization. J. Mod. Power Syst. Clean Energy.

[9] Wang W, Yuan B, Sun Q, Wennersten R (2022). Application of energy storage in integrated energy systems—A solution to fluctuation and uncertainty of renewable energy. J. Energy Storage.

[10] Ali Dashtaki A, Mehdi Hakimi S, Hasankhani A, Derakhshani G, Abdi B (2023). Optimal management algorithm of microgrid connected to the distribution network considering renewable energy system uncertainties. Int. J. Electr. Power Energy Syst..

[11] Kanakadhurga D, Prabaharan N (2022). Demand side management in microgrid: A critical review of key issues and recent trends. Renew. Sustain. Energy Rev..

[12] Mota B, Faria P, Vale Z (2022). Residential load shifting in demand response events for bill reduction using a genetic algorithm. Energy.

[13] Praveen M, Rao GVS (2020). Ensuring the reduction in peak load demands based on load shifting DSM strategy for smart grid applications. Procedia Comput. Sci..

[14] Urbanucci L (2018). Limits and potentials of mixed integer linear programming methods for optimization of polygeneration energy systems. Energy Procedia.

[15] Dokeroglu T, Sevinc E, Kucukyilmaz T, Cosar A (2019). A survey on new generation metaheuristic algorithms. Comput. Ind. Eng..

[16] Hp C, Subbaramaiah K, Sujatha P (2023). Optimal DG unit placement in distribution networks by multi-objective whale optimization algorithm & its techno-economic analysis. Electr. Power Syst. Res..

[17] Yehia M, Allam D, Zobaa AF (2022). A novel hybrid fuzzy-metaheuristic strategy for estimation of optimal size and location of the distributed generators. Energy Rep..

[18] Reddy GH, Koundinya AN, Gope S, Raju M, Singh KM (2022). Optimal sizing and allocation of DG and FACTS device in the distribution system using fractional lévy flight bat algorithm. IFAC-PapersOnLine.

[19] Djidimbélé R, Ngoussandou BP, Kidmo DK, Kitmo BM, Raidandi D (2022). Optimal sizing of hybrid systems for power loss reduction and voltage improvement using PSO algorithm: Case study of Guissia Rural Grid. Energy Rep..

[20] Fathi R, Tousi B, Galvani S (2023). Allocation of renewable resources with radial distribution network reconfiguration using improved Salp Swarm Algorithm. Appl. Soft Comput..

[21] Ang, S., Chhor, U., Chayakulkheeree, K. & Ieng, S. *Grey Wolf Optimizer for Optimal Allocation and Sizing of Distributed Generation for Loss Reduction and Voltage Improvement in Distribution System Optimal Power Flow Considering Price-Based Real-Time Demand Response View Project Power Economic Dispatch; Power Optimization View Project*. https://www.researchgate.net/publication/362015260 (2022).

[22] Rawa M, Abusorrah A, Bassi H (2021). Economical-technical-environmental operation of power networks with wind-solar-hydropower generation using analytic hierarchy process and improved grey wolf algorithm. Ain Shams Eng. J..

[23] Trojovska E, Dehghani M, Trojovsky P (2022). Zebra Optimization Algorithm: A new bio-inspired optimization algorithm for solving optimization algorithm. IEEE Access..

[24] Zhao S, Zhang T, Ma S, Wang M (2022). Sea-horse optimizer: A novel nature-inspired meta-heuristic for global optimization problems. Appl. Intell..

[25] Hemeida MG, Alkhalaf S, Senjyu T, Ibrahim A, Ahmed M, Bahaa-Eldin AM (2021). Optimal probabilistic location of DGs using Monte Carlo simulation based different bio-inspired algorithms. Ain Shams Eng. J..

[26] Janamala V (2021). A new meta-heuristic pathfinder algorithm for solving optimal allocation of solar photovoltaic system in multi-lateral distribution system for improving resilience. SN Appl. Sci..

[27] Javad AM, Radmehr M (2021). Optimization of hybrid renewable energy system in radial distribution networks considering uncertainty using meta-heuristic crow search algorithm. Appl. Soft Comput..

[28] Akbar MI, Kazmi SAA, Alrumayh O, Khan ZA, Altamimi A, Malik MM (2022). A novel hybrid optimization-based algorithm for the single and multi-objective achievement with optimal DG allocations in distribution networks. IEEE Access..

[29] Khan MH, Ulasyar A, Khattak A (2022). Optimal sizing and allocation of distributed generation in the radial power distribution system using honey Badger algorithm. Energies (Basel).

[30] Naderipour A, Nowdeh SA, Saftjani PB (2021). Deterministic and probabilistic multi-objective placement and sizing of wind renewable energy sources using improved spotted hyena optimizer. J. Clean Prod..

[31] Montoya OD, Gil-González W, Orozco-Henao C (2020). Vortex search and Chu–Beasley genetic algorithms for optimal location and sizing of distributed generators in distribution networks: A novel hybrid approach. Eng. Sci. Technol. Int. J..

[32] Radosavljevic J, Arsic N, Milovanovic M, Ktena A (2020). Optimal placement and sizing of renewable distributed generation using hybrid metaheuristic algorithm. J. Mod. Power Syst. Clean Energy.

[33] Rafi V, Dhal PK (2020). Maximization savings in distribution networks with optimal location of type-I distributed generator along with reconfiguration using PSO-DA optimization techniques. Mater. Today Proc..

[34] Olatunde O, Rahman HA (2019). Allocation of distributed generation and capacitor banks in distribution system. Indones. J. Electr. Eng. Comput. Sci..

[35] Sridhar JP, Prakash R (2019). Multi-objective whale optimization based minimization of loss, maximization of voltage stability considering cost of DG for optimal sizing and placement of DG. Int. J. Electr. Comput. Eng..

[36] Ali ZM, Aleem SHEA, Omar AI, Mahmoud BS (2022). Economical-environmental-technical operation of power networks with high penetration of renewable energy systems using multi-objective coronavirus herd immunity algorithm. Mathematics.

[37] Zakariazadeh A, Jadid S, Siano P (2014). Stochastic multi-objective operational planning of smart distribution systems considering demand response programs. Electr. Power Syst. Res..

[38] Willy Online Pty Ltd. https://wind.willyweather.com.au/.

[39] Grigg, C. *et al*. *The IEEE Reliability Test System = 1996 Application of Probability Methods Subcommittee A Report Prepared by the Reliability Test System Task Force of the Figure 1-IEEE One Area RTS-96*, Vol. 14 (1999).

[40] Nasir T, Bukhari SSH, Raza S (2021). Recent challenges and methodologies in smart grid demand side management: State-of-the-art literature review. Math. Probl. Eng..

[41] Bertineti, D. P., Canha, L. N., Medeiros, A. P., de Azevedo, R. M. & da Silva, B. F. *Heuristic Scheduling Algorithm for Load Shift DSM Strategy in Smart Grids and IoT Scenarios*.

[42] Javaid N, Hafeez G, Iqbal S, Alrajeh N, Alabed MS, Guizani M (2018). Energy efficient integration of renewable energy sources in the smart grid for demand side management. IEEE Access..

[43] Khalid A, Javaid N, Guizani M, Alhussein M, Aurangzeb K, Ilahi M (2018). Towards dynamic coordination among home appliances using multi-objective energy optimization for demand side management in smart buildings. IEEE Access..

[44] Tang H, Wang S, Li H (2021). Flexibility categorization, sources, capabilities and technologies for energy-flexible and grid-responsive buildings: State-of-the-art and future perspective. Energy.

[45] Papazoglou G, Biskas P (2023). Review and comparison of genetic algorithm and particle swarm optimization in the optimal power flow problem. Energies.

[46] Grisales-Noreña LF, Montoya OD, Gil-González W (2019). Integration of energy storage systems in AC distribution networks: Optimal location, selecting, and operation approach based on genetic algorithms. J. Energy Storage.

[47] Zheng Y, Song Y, Hill DJ (2020). A general coordinated voltage regulation method in distribution networks with soft open points. Int. J. Electric. Power Energy Syst..

[48] Rana, A. D., Darji, J. B., Pandya, M., Student, P. G., Department, E. E. & Bad, A. *Backward/Forward Sweep Load Flow Algorithm for Radial Distribution System*, Vol. 2. www.ijsrd.com (2014).

[49] Nassef AM, Abdelkareem MA, Maghrabie HM, Baroutaji A (2023). Review of metaheuristic optimization algorithms for power systems problems. Sustainability.

[50] Dewangan CL, Singh SN, Chakrabarti S, Singh K (2022). Peak-to-average ratio incentive scheme to tackle the peak-rebound challenge in TOU pricing. Electr. Power Syst. Res..

